# What is built and what is taught: The difference between teaching and professional practice in building structures

**DOI:** 10.1007/s44150-022-00056-7

**Published:** 2022-06-24

**Authors:** Carlos Olmedo, Alejandro Calle, Joaquín Antuña

**Affiliations:** Departamento de Estructuras y Física de Edificación, Universidad Politécnica de Madrid, Juan de Herrera, 4, Madrid, 28040 Madrid, Spain

**Keywords:** Building structures, Teaching building structures, Structures in architecture

## Abstract

The authors show the great difference that exists between structural solutions that are taught and that students have to resolve to obtain the qualification of Architect and the constructive and structural solutions that are common in professional practice in Spain. A database of structural solutions used in master’s degree projects for professional qualification and their results was therefore prepared and compared with existing statistical construction data. It can be deduced from this data that there is a discrepancy between students’ proposals and usual practice, in structural types as well as in terms of size, materials and building category.

## Introduction

The regulation of the profession of architect in Spain requires the qualification of a specific master’s degree in architecture open only to those who already hold a bachelor’s degree in architecture.

In accordance with Spanish legislation, this master’s degree provides the necessary professional skills that allow an architect —without the intervention of any other technician— to undertake any building project.

Building structure is one of the tasks which has to be defined for this. Teaching architecture, therefore, involves mechanics, strength of materials and the theory of structures, all of which may account for up to 20% of studies. To be able to be fully qualified to exercise the regulated profession it is necessary to obtain the 300 credits corresponding to the degree in *Fundamentos de la Arquitectura* and the 60 credits corresponding to the master’s degree in architecture. The master’s degree includes undertaking a building project which includes all the documents that are necessary for a professional project. An obligatory 5-credit subject centres on resolving the structural part of the project. The authors, who have taught this subject for the last six years in the *Escuela Técnica Superior de Arquitectura de Madrid* (ETSAM), have prepared a summary of the structural solutions used in the projects used by the students, and they compared them with structures built in Spain during the past 10 years.

The question raised revolves around two interrelated points regarding how to teach architecture. The first is about the desirability of including technical content in studies, and if the answer is positive, what should its focus be. The second point derives from the first, and consists of how structures should be taught. Studying students’ projects has allowed to identify two relevant aspects. On the one hand, most proposals are hardly feasible because of their size or technical challenges. On the other, their proposed constructive and structural solutions do not conform to usual building practices in Spain. The former point raises doubts about project sustainability, as the generalization of such constructions would increase building industry contributions to greenhouse gas emissions, opposing United Nations sustainable development goals. The latter casts questions about the efficacy of what is taught.

## Origin of the qualification of Architect in Spain

Architecture was taught in Spain by the *Academia de Bellas Artes de San Fernando* from its creation in 1752. Copying and drawing models formed the main part of the learning program. Although scientific and technical subjects were included, they were taught in the first years and were not a relevant part of the training. The technical shortcomings of the architects trained in this way were evident, and several projects to renovate the study plans were examined [1, 2].

The situation changed after 1844, when the *Escuela Superior de Arquitectura* was created. It started work in the academic year of 1845-1846 and, with several changes, is still the institution where architects learn their trade. The curriculum changed in several ways during the XX century, although in all cases preparatory courses were included, in which scientific and technical training was given. Although these courses were taught for some years in a Preparatory School, they were eventually taught in the Science Faculty. These courses were shared by architects and civil and mining engineers [3].

In the 1914 curriculum, the following first-year subjects were taught in the Science Faculty: arithmetic and basic algebra, higher algebra, geometry and trigonometry, analytical geometry, physics, chemistry and mineralogy. Once students had entered the school there were two introductory courses covering calculus, descriptive geometry and rational mechanics, together with copying ornamental elements, the history of art, modelling and architectural details [4].

In 1957, a series of legislative changes reformed technical education. Two selective courses were kept during the first years, the first of which was common to all of the technical subjects, engineering and architecture. In 1964 the courses prior to admission were eliminated, although the subjects of basic science and drawing which had been included in the previous courses remained. In the following years, and in the context of a debate centring on the teaching of architecture and its role in society, in which students participated, there was a demand to include scientific and technical contents in how projects were prepared [5].

## The current qualification of Architect in Spain and in ETSAM

The architecture curricula used now in Spain date from 2010, when new curricula were introduced in the different schools to adapt to the European Higher Education Area. There is an initial bachelor’s degree in architecture, which lasts for ten terms and ends with a final degree work. This is all worth 300 credits of the European Credits Transfer System [6]. It is indispensable to have passed this degree course to be able to access the master’s degree in architecture course, which is worth 60 credits.

In Spain there are 25 recognized master’s degrees in architecture and ETSAM graduates represent almost 25% of all architects who graduate in the whole country (Table 1).

**Table 1:** Master’s degrees in architecture in Spain. Students enrolled and graduated in the 2019-2020 academic year. Source: statistical request answered by the Spanish Ministry of Universities: https://www.universidades.gob.es/portal/site/universidades/menuitem.b96568fef1ce8b35c7d86f10026041a0/?vgnextoid=cfb0372673680710VgnVCM1000001d04140aRCRD

		Students	
University	School	enrolled	graduate
	Public universities	2.009	1.272
A Coruña	Escuela Técnica Superior de Arquitectura	63	26
Alcalá	Escuela de Posgrado	78	40
Alicante	Escuela Politécnica Superior	57	28
Girona	Escuela Politécnica Superior	40	40
Granada	Escuela Internacional de Posgrado	91	69
Málaga	Escuela Técnica Superior de Arquitectura	37	16
País Vasco	Escuela Técnica Superior de Arquitectura	86	56
Politécnica de Cartagena	Escuela Técnica Superior de Arquitectura y Edificación	4	4
Politècnica de Catalunya	Escuela Técnica Superior de Arquitectura	72	60
	Escuela Técnica Superior de Arquitectura del Vallés	154	89
Politécnica de Madrid	Escuela Técnica Superior de Arquitectura	577	340
Politècnica de València	Escuela Técnica Superior de Arquitectura	349	221
Rey Juan Carlos	Escuela de Másteres Oficiales	48	37
Sevilla	Escuela Técnica Superior de Arquitectura	243	163
Valladolid	Escuela Técnica Superior de Arquitectura	64	50
Zaragoza	Escuela de Ingeniería y Arquitectura	46	33
	Private universities	130	99
Antonio de Nebrija	Escuela Politécnica Superior	28	12
Europea de Canarias	Escuela de Arquitectura	10	7
Europea de Madrid	Escuela de Arquitectura, Ingeniería y Diseño.	19	12
Europea de Valencia	Escuela de Arquitectura y Politécnica	12	7
Navarra	Escuela Técnica Superior de Arquitectura. Campus de Madrid	54	54
Ramón Llull	Escuela Técnica Superior de Arquitectura La Salle	7	7

### Degree in architectural fundamentals

The general teaching structure of the ETSAM degree in architectural fundamentals is organised in three modules: preparatory module, technical module and project module. The subjects which specifically correspond to structures are covered in the technical module, in five obligatory subjects each one of which lasts for one term, with a total of 30 ECTS. In the second, third and fourth year the basic theory of structures and strength of materials are covered in “Structures 1”, “Structures 2” and “Structures 3”, and in the fifth year, as well as studying soil mechanics and foundations, there is a subject denominated “Structures Project”.

In addition to the compulsory subjects, students can choose up to 24 more credits in structures by choosing the optional workshops offered.

### Authorising master’s degree

In the ETSAM master’s degree in architecture, the students prepare a project for a building or set of buildings with the aim of gaining professional competence. This includes the development of the structural part of the building, as well as the dimensioning of the different installations that are necessary and the resolution of the constructive needs of the whole building. The master’s degree is organised in four blocks and divided into two terms, each one of which covers different aspects of the project:

- Technical module: 8 obligatory ECTS
- Project module: 12 obligatory ECTS
- Architectural intensification/research module: 10 optional ECTS
- End of master’s degree work (end of course project [PFC]): 30 obligatory ECTS

In the first term of the master’s degree course, teaching is organized around project workshops, composition, and town planning, and the aim is to define the formal and functional organisation of a building, at a basic professional project level. In the second term, the structures and construction workshops go on to define the building in detail [7].

The structures workshop lasts for nine weeks in the second term. The specific skills of the end of course project module are [7]:

- Aptitude to conceive, analyse, design and integrate structures in buildings and urban projects.
- Aptitude to project and execute and develop construction management.
- Aptitude to project interventions in existing buildings and their maintenance and rehabilitation.
- Once the other credits for the curriculum have been obtained, students have to prepare, present and defend an original exercise undertaken individually before a university jury which has to include at least one professional with recognised prestige nominated by professional organisations. The exercise will consist of a complete and professional architectural project which brings together all the skills acquired during the degree course, at a standard high enough to demonstrate the capacity to determine the complete execution of the building works in question, complying with the applicable technical and administrative regulations.

### Authorising master’s degree structures workshop organisation

In the structures workshop, the students have to prepare a document which proves that the building fulfils the structural requisites of strength, stiffness and balance as stipulated by the applicable regulations, including the graphic documentation that is necessary for the construction of the structural part of the building. The specific skills corresponding to the structures workshop are [7]:

Aptitude for conceiving, calculating and designing structures in buildings and urban settings, and executing building structures.

## Description of research

A total of 171 student projects were analysed, distributed over two terms of academic year 2019/2020 and the first term of academic year 2020/21 (during half of this time, the works analysed were prepared during the COVID-19 pandemic, and the workshop was online). They were prepared in the structures workshop by students in seven different teaching units. The authors estimate that the time involved and the range of teaching units analysed, make the sample sufficiently representative.

A database was created from these submissions that includes the definition of a series of structural properties. These include the definition and justification of the structural solution they propose, with complete plans and the necessary details, as well as the justificatory report with its calculation appendix.

The database holds an amount of data ranging from general items such as title, location and use, to more specific matters about the structure, classified under five headings: foundations and exterior walls, horizontal structure, vertical structure, bracing system, and roofing system.

Each of these sections includes several groups with the particular solutions used in each case, to discover how often each one of the said systems is used.

To compare the data obtained with actual buildings in Spain, official data from the *Ministerio de Transportes, Movilidad y Agencia Urbana* were used. These data are from a questionnaire that has to be completed for each work that is built, which has to be processed by the ministry. This questionnaire is broad and covers different data, from the work developer, its location or planned duration, up to its use and the types of building techniques used.

In our study we wanted to gather information on the type of building and the constructive system used. Although the classification system used in the questionnaire is far less detailed than the one we used to describe the end of master’s degree course projects, the types defined in the questionnaire can be linked to those we identify in the study of teaching projects.

**Fig. 1:**
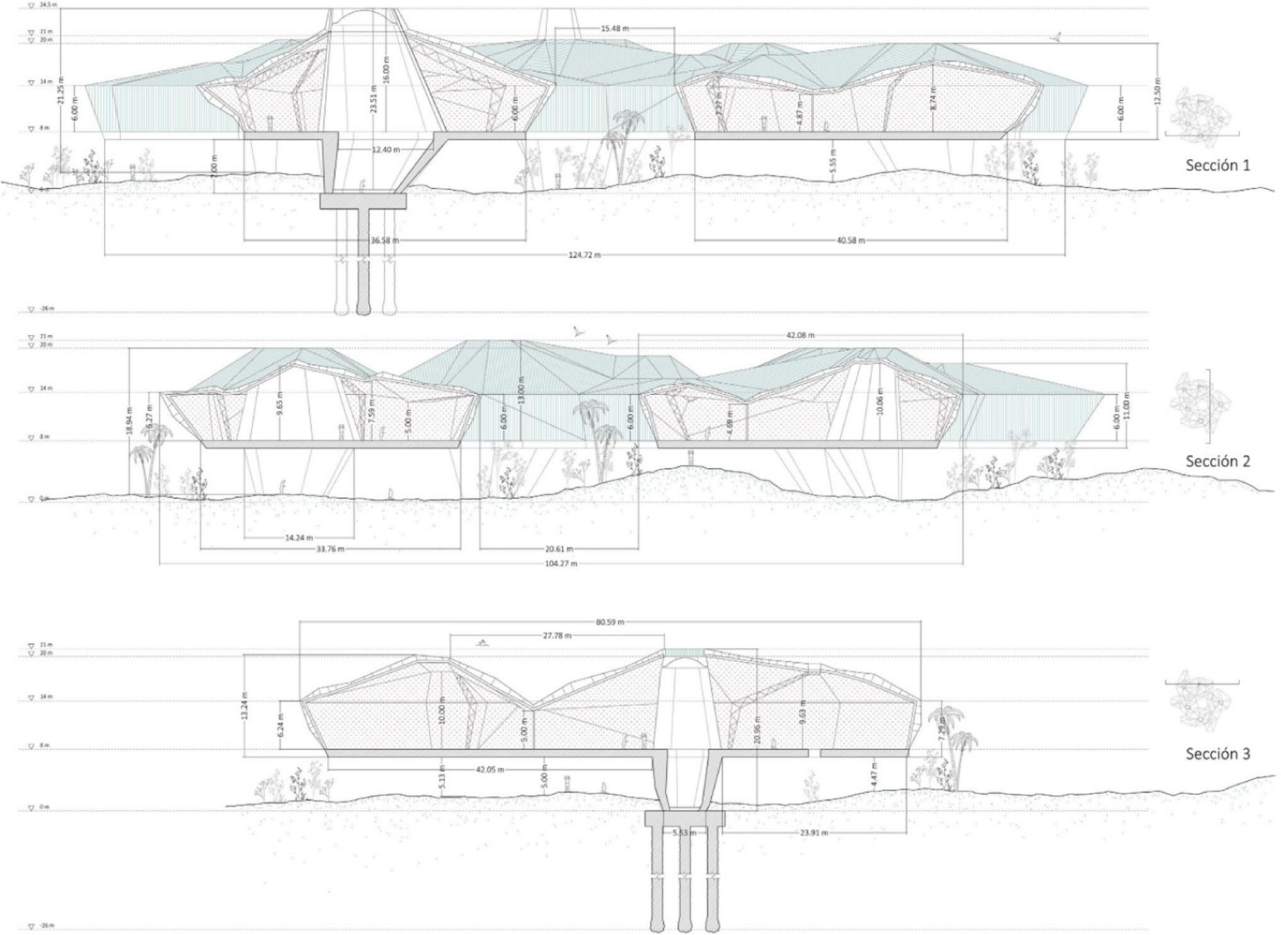
Student project: María Iglesias Alperi, 2019-2020

The following tables link the qualities identified in the students’ works with the ones defined in the questionnaire. As may be seen, the description of the structural types used in the projects is far more extensive than the one used in the questionnaire. The latter simply considers five types which also describe the whole building. On the other hand, although it is clear in some cases which type the authors may have included in the questionnaire, such as a structure using beams, supports and reinforced concrete floor, this is often not the case. The following tables do not aim to show an exact correspondence between the types used in the teaching projects consulted and those included in building and housing statistics (Tables 2, 3, 4, and 5; Fig. 1).

**Table 2:** Types of horizontal structure identified in master’s degree end-of-course projects, and the types identified in building statistics

Structure use in teaching projects	Structure in the survey
Horizontal structure, main beams	
Reinforced concrete shallow beams	Reinforced concrete
Deep beams	
Prefabricated prestressed concrete beams	
Steel beams	Steel
Composite beams	Composite
Timber beams	Others
Horizontal structure, floor	
Cast-in situ girders	Reinforced concrete
Reinforced tie-beams	
Post-tensioned beams	
Timber purlins	Others
Steel joist	Steel
Prestessed plates	Reinforced concrete
Pre–slabs	
Double tee joist	
CLT floor	Others
Precast prestressed concrete beams	Composite
Slab	Reinforced concrete
Post-tensioned slab	
Waffle slab	
“Bubble deck”

**Table 3:** Types of vertical structure identified in master’s degree end-of-course projects, and the types identified in building statistics

Vertical structure	
R. C. columns	Reinforced concrete
Precast R. C. columns	
Steel columns	Metal
Steel struts	
Laminated wood columns	Others
Masonry	Masonry
Reinforced concrete walls	Reinforced concrete
CLT walls	Others

**Table 4:** Types of bracing systems identified in master’s degree end-of-course projects, and the types identified in building statistics

Bracing systems	
Rigid frame	Reinforced concrete
Rigid frame	Steel
Semi–rigid MLE joints	Others
Steel struts	Metal
Steel cables	
Masonry	Masonry
Reinforced concrete walls	Reinforced concrete
CLT walls	Others

**Table 5:** Types of roofing systems identified in master’s degree end-of-course projects, and the types identified in building statistics

Roofing systems	
Steel plane frames	Steel
Flat wooden roof frames	Others
Spatial steel roof frames	Metal
Spatial wooden roof frames	Others
Textile structures	Others
Pneumatic structures	Others
Vault or dome systems	Others
Others	Others

## Types of structure in master’s degree projects

**Fig. 2:**
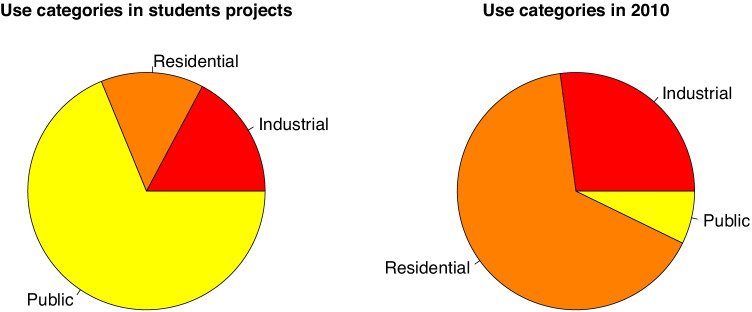
Distribution of uses of the buildings in educational projects (left) and in the building industry in 2010 (right)

The use for which a building is designed, even though it is not a structural characteristic, is a relevant datum when deciding which type of constructive solution to use. Three groups are defined: housing, industrial and public access. Results show that the uses for the purposes of master’s degree projects are public use, at 63% of the total, 15% for industrial use and only 12% for residential use (see Fig. 2). Almost three quarters of the buildings constructed are for housing, while public buildings represent only a tenth of the total.

This distribution will give us an idea of the range of types of structure and design solutions that may be found in educational projects. Residential usage has certain requisites which have to be respected, and this largely limits the available solutions. There is a similar situation in industrial uses, although the wide range of industries means that here there are more different types of buildings. Nevertheless, public buildings include a wide variety of uses: educational, medical or recreational, etc., so that a wider range of constructive and structural types may be proposed here.

Structural types used for the master’s degree final project, organised in five parts, are now described: foundations, floor structure, vertical structure, bracing system, roofing structure. The structural solution of each part was recorded for each project.

### Types of foundations

**Fig. 3:**
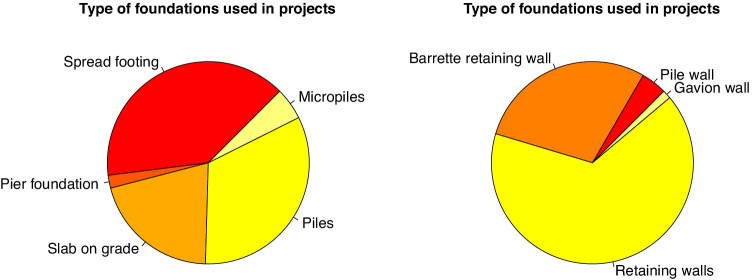
Types of foundations (left) and retaining walls (right) found in student educational projects

Foundations are grouped into two main categories: footings (that resist vertical loads) and retaining walls (that resist earth lateral pressure). The most common system is to extend footings under columns or walls, and these are present in almost half of the projects. The other half uses piles or foundation slabs. For earth pressure containing, the most common solution is retaining walls, while a quarter are piles or barrette walls (see Fig. 3).

### Floor structure solutions

This section includes structural solutions for flooring (horizontal structures within internal spaces). Three broad types may be used (reinforced concrete, steel and wood) a more detailed description is given to specify the type of solution used.

**Fig. 4:**
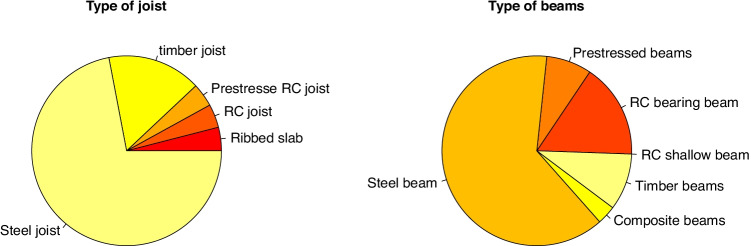
Type of horizontal structure used in student projects. Beam and joist solutions have been separated. Steel is the most common material

**Fig. 5:**
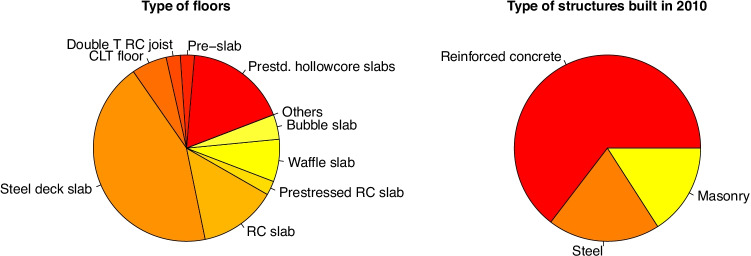
Flooring types used in master’s degree projects (left) and in buildings in 2010 (right)

Three elements were used to describe horizontal structure: the flooring system, joist type and beam type. This way all of the systems used in the projects can be described. The most widely used flooring system consists of steel deck slabs (45% of cases). The others use different reinforced concrete solutions, most often slabs and hollow core slabs (20% each) and the remainder use other reinforced or prestressed concrete systems (see Fig. 4). 75% of joists and 66% of beams are made of steel. This difference arises because in many cases steel joists are used to support concrete slabs or timber panels. After steel, the most widely used material for joists is timber, which is used in 15% of cases. The same proportion applies to timber beams (see Figs. 4 and 5).

### Vertical structure solutions

**Fig. 6:**
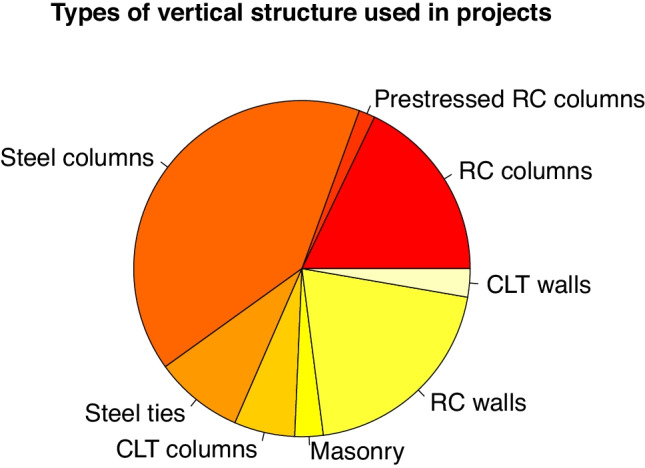
Type of vertical structure use in students’ projects

**Fig. 7:**
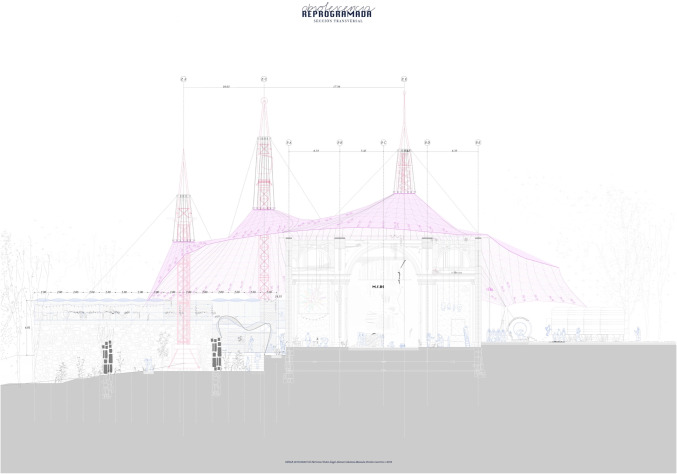
Student project: Manuela Perales, 2020-2021

Different vertical structure solutions were identified in the projects studied. RC columns are used in 27% of the cases, including cast-on-site and precast solutions. Steel columns were used in 57% of all the solutions. The cases in which steel struts were used to support suspended sections of floor have also been shown. In general, this solution is associated with the use of steel columns and beams, and it is used in 12% of the projects that were studied, although those that use steel columns are also included here. Laminated wood columns are used in 8% of cases, and CLT walls are used in 4%. Lastly, stonework walls are used in 4% of cases (see Figs. 6 and 7).

### Bracing system

**Fig. 8:**
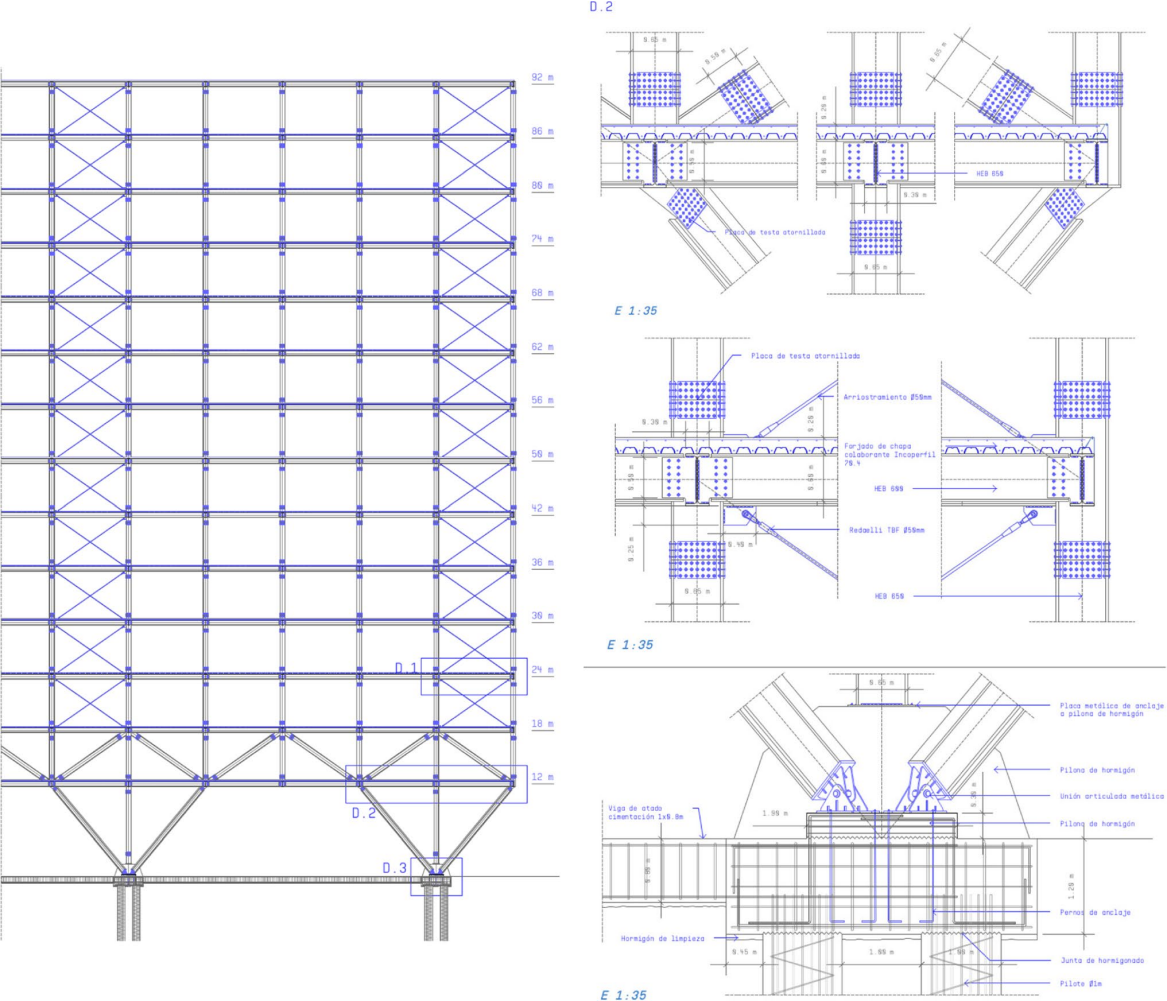
Student project: Álvaro Mesquida, 2020-2021

Stability is a fundamental aspect of structural design, and it has to be taken into account from the start of a project. In those cases where structures consist of beams and reinforced concrete supports joined by rigid connections, correct dimensions will ensure stability. However, in other types of structure it may be impossible to achieve this, making it necessary to modify the overall design. This is why it is one of the aspects which is emphasised the most in the workshop (Fig. 8).

**Fig. 9:**
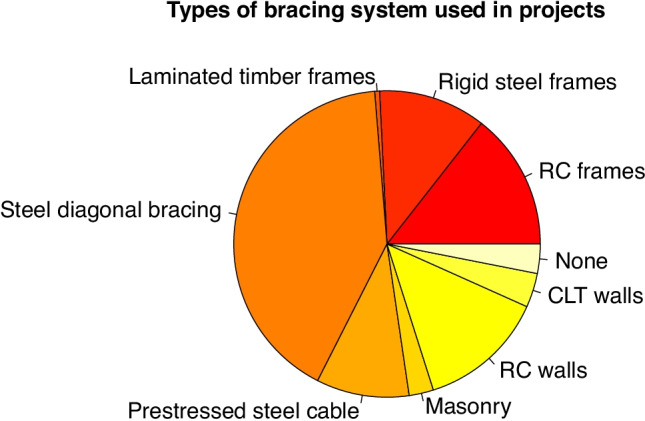
Bracing systems uses in students’ projects in order to ensure stability

A total of eight types of solutions were identified. Frames of reinforced concrete beams and columns were used in 14% of the solutions, while steel portal frames with rigid joints were used in 11% and semi-rigid joints were used in 1%. Bracing by diagonal struts was used in 41% of cases, cables were used in 10% and masonry walls were used in 3% of the total, while reinforced concrete walls were used in 13% and CLT walls were used in 4% of cases. Only 3% of the projects did not include any bracing (see Fig. 9).

### Roofing systems

In many types of building the roof is a differentiated element, with a structural form different from that of the rest of the building. For example, in a conventional block of flats, the roof is a floor structure of the same kind as the other floors, solely differentiated by the magnitude of the loads which it supports. Nevertheless, in buildings which have other uses, the roof can become their identifying element. Given the large number of public buildings which are planned as project exercises, in many of them the roof is the differentiating element of the project.

**Fig. 10:**
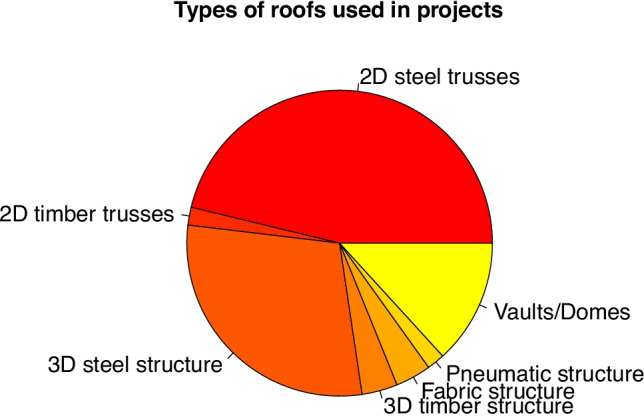
Roofing systems uses in students’ projects

To resolve special roofs, generally with a span greater than the others in a project, the type of structure used the most often consists of steel trusses (46%) or another type of special structure in steel (29%). However, wooden trusses are also used (2%) or special structures in wood (4%), as well as textile roofs (4%), inflatable structures (2%) or vaults (13%) (see Fig. 10).

## Types of structure in constructed projects

**Fig. 11:**
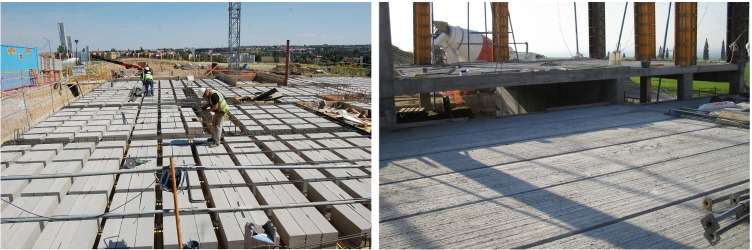
Almost two thirds of buildings in Spain are built with a system similar to the one shown on the left: a set of secondary beams separated by lightening blocks and supported by a set of main beams. Pre-cast systems can also be used, such as the hollow core slabs of the image at the right

Thanks to the building and housing statistics managed by the *Ministerio de Transportes, Movilidad y Agenda Urbana*, it is possible to know the number and types of construction which are built per year in Spain [8] (Fig. 11).

**Fig. 12:**
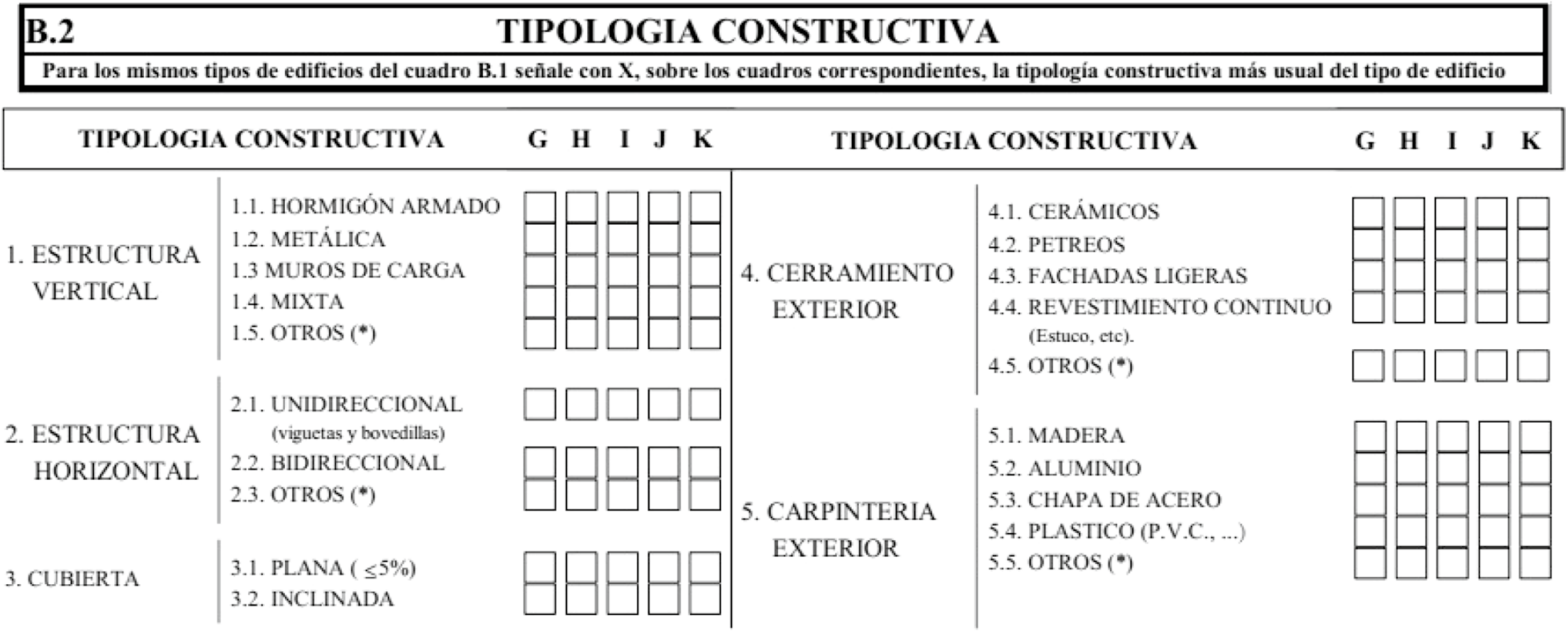
Part of the survey by the *Ministerio de Transportes, Movilidad y Agenda Urbana*, showing data it collected. Point 1 and 2 refer to the building structure. 1 shows five options for vertical structure: reinforced concrete, steel, masonry, steel decking and others. 2 contains three types of floor system for horizontal structure: single slab, bi directional slab or others

This survey contains several types of data. We searched for information on building use, in terms of the number of constructions by use and the surface area they contain. Constructive structural aspects included in the questionnaire are simple and broader in scope than the concepts used to describe the master’s degree projects. Nevertheless, the data given make it possible to gain a clear idea of the types of structure that are built in Spain. Figure 12 shows the section of the questionnaire that includes project structure data.

**Fig. 13:**
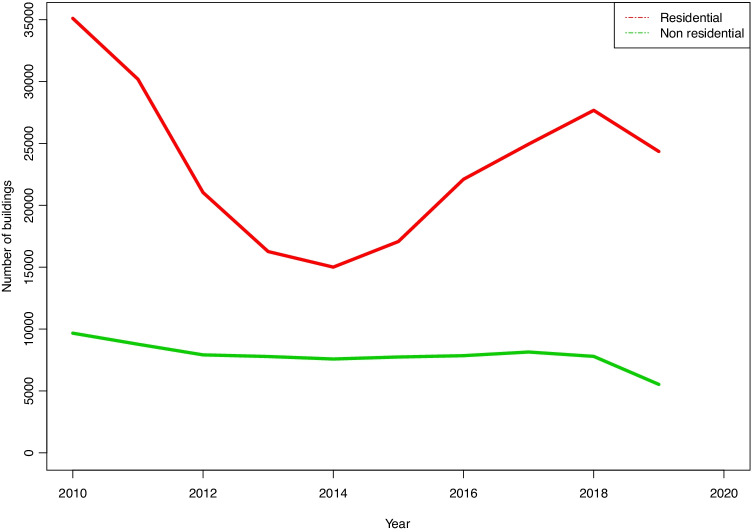
Total number of buildings in last ten years

Two sections coincide with two of the ones considered when studying the master’s degree projects: vertical and horizontal structure. Although the content of each one of these sections is smaller than the content used with the student’s projects, it makes it possible to clearly group the solutions that are used. Thus with reference to the vertical structure, the proposed types agree with the ones studied, and the lack of wood as an option may be the clearest absence. This situation is perfectly comprehensible and explicable in Spain, where to date wood has hardly been used in building (Fig. 13).

Respecting horizontal structure, the questionnaire seems to solely recognise concrete floors, leaving all of the other possible systems to be included under the generic term “Others”. This group would contain mixed structures as well as wooden floor structures.

**Fig. 14:**
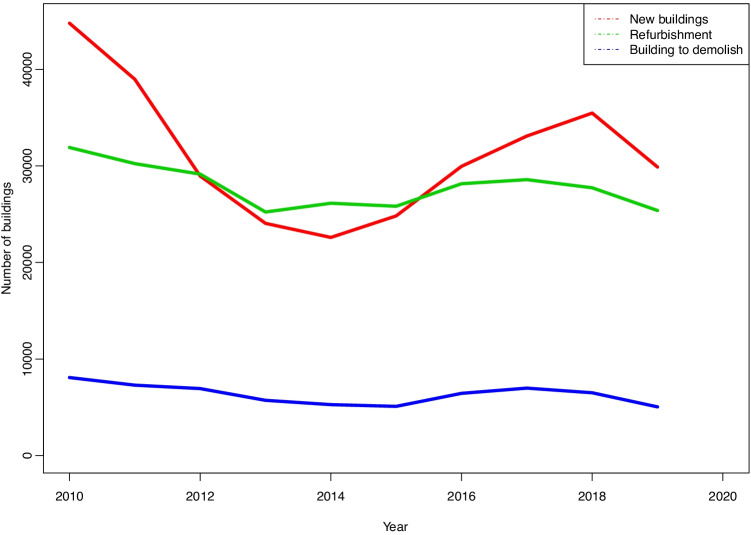
New buildings and refurbishment

Data from the last ten years have been selected to represent building activity. The questionnaire asks about other building data. It consists of six pages, and one of them is used to describe renovation works. This is a highly important datum, as during the years covered the number of new buildings is similar to the number of renovation works (Fig. 14).

Another relevant item of information, although it does not refer directly to the structure of projects, is planned use. In this respect, the variation over recent years shows that the largest number of buildings are for residential use and that the proportion of these within the total may vary from more than 70% in 2010 to 60% around 2014.

**Fig. 15:**
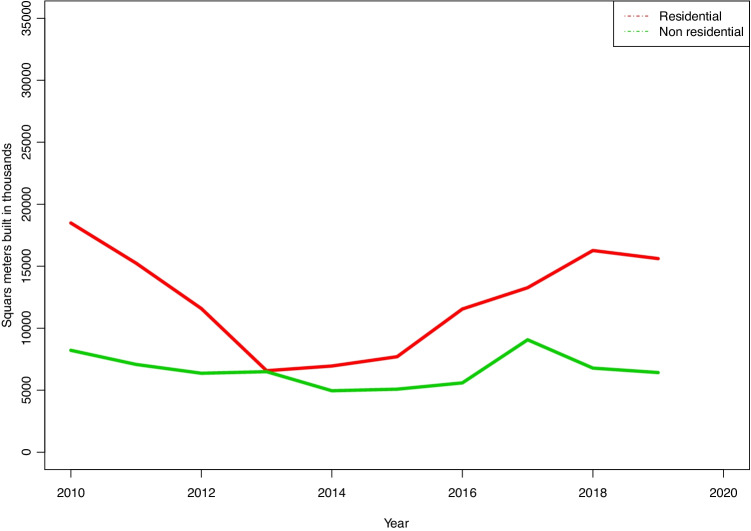
Built surface areas for different uses

These first two items of data give a clear idea of building activity: approximately half of the projects are for renovation, while the other half consists of new buildings. Of the latter, housing varies from 60% to more than 75% in terms of the number of works and built surface area, while housing represent from 50% to 60% of the total amount of work (Fig. 15).

Lastly, it can be seen in the classification of vertical structures that reinforced concrete construction is clearly more common than any other type, in a proportion that may vary from 60% to 80% of the total, depending on the year.

**Fig. 16:**
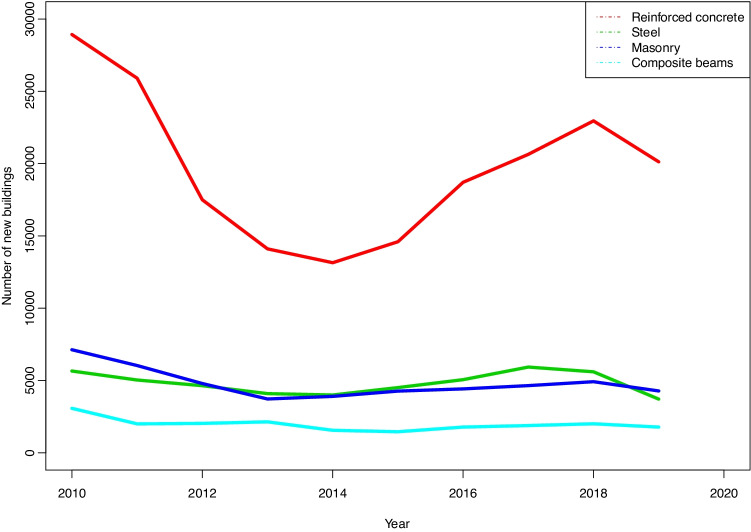
Types of material used in built structures

The next outstanding point is that the number of works undertaken using stonework is similar to the number made using steel, and that the proportion varies from 10% to 20%, depending on the year (Fig. 16).

Finally, all other types of structure built would fit under the heading “mixed construction”, as no other heading would be suitable for them.

## Discussion

Based on the data analysed it is possible to underline the following differences between the structural part of the students’ projects and the structures which are actually planned and built:

- Although it does not specifically refer to structural solutions, the difference in uses stands out in the first place as it is relevant to the type of solution selected. According to the building statistics, more than 70% of projects are for housing, and, in terms of the built surface area, more than 60% of this is for the same purpose. The corresponding figure for master’s degree projects is less than 12% of the total.
- Respecting the type of work, according to the building statistics the number of projects for new buildings is similar to the number of renovation work projects. Although in this case there is no reference to the built surface area for each type, it may be foreseen that the surface area renovated will be smaller than the surface area of new buildings. Nevertheless, the difference here in comparison with the master’s degree projects is significant, as hardly 1% of the latter consist of renovation work.
- A very high percentage of the students’ projects are for very large buildings, while the average surface area of a “real” project stands at 600 m^2^.
- The large size of the projects means in the majority of cases that structural elements have to cover large spans which often consist of large-scale engineering work.
- Respecting the materials used, the majority of the students’ projects avoid the use of “domestic” techniques and materials, as they prefer other solutions. Once again, this may be due to the size of their projects, which make more “technological” solutions necessary in the use of materials, too. This may give rise to graphic documentation that is more “striking” or more appealing to modern tastes. In the case of what is actually built, most works use concrete in ”one-way” type structures in floors. It is interesting to note that there are practically no stonework structures in the students’ projects, although in actual buildings it is still relatively common —although it is surely restricted to structures in detached houses—, with a similar number of projects that use this material to those built using steel.

To understand some of the reasons for this difference, the fact that many architecture students are more interested in the “human” side of the discipline than they are in technical questions should be taken into consideration [9]. Moreover, for structures, teaching programs generally place more emphasis on analysis than they do on conceptual structural design [10]. As a consequence, there is a widespread belief that analysis is able to resolve “everything”, and that it is therefore possible to forget that structural design is a part of the architectural project, as it will be resolved instantaneously with the help of a computer program, given that according to this stance, it is “only” a calculation with a single result. Structure is then often thought to be something which is “stuck” onto architecture, rather than something which is a part of it [11].

As teachers we have found that there is common belief that structure can be added as an artefact to any architectural project using software in the last phase of the design, allowing students to suppose that they can handle very large and complex projects, applying substantial changes to it until the very last moment without having to worry too much about structure. Moreover, most of the structural solutions, if they were to be executed in a full construction project, would need to be undertaken in collaboration with a multidisciplinary team working exclusively on the structural solution, often with innovative solutions. However, students have to be able to do this work individually, and it is sometimes tremendously difficult. The work involved is highly intense, and the resulting learning process is usually evident. However, the results when a proposed solution is very specific may be exclusively applicable to the proposal in question.

The question of size illuminates the contradiction between the focus on sustainability —which is assumed to be adopted not only in academia, but also as an obvious need throughout society— and the selection of such large projects, which involve very heavy resource consumption. As a whole, the construction sector, taking into account the construction and maintenance of buildings, accounts for 38% of greenhouse gas emissions; regarding energy consumption, it represents 35% of total consumption. More specifically, 55% of the cost of electrical energy consumption is due to buildings [12]. Reducing energy consumption and emissions both in construction and in the use of buildings is therefore the main challenge for our society. In the case of structure, although it does not represent more than 20% of total cost, a fundamental objective should be to make structures that use materials with the lowest energy cost and the smallest possible amount of materials. To achieve this, the authors believe it should be essential to start with the education of the technicians who will have to develop new construction techniques in the future.

As teachers, we should be able to include this major issue in structure courses. Size, materials, cost and sustainability should be the pillars of structural design rather than a cosmetic add-on. In this case, projects should take the sustainable use of resources into account, applying the same level of exigence as is the case for resistance or stiffness. This reasoning should obviously be extended to the whole project process.

To return to the discussion regarding the gap between teaching and professional practice, we should ask ourselves if this is not actually the best situation, given that academia is not and will never be practice. There always has been a difference between what is taught at university and what is relevant in practice. The authors believe that higher education should never consist of “high-level professional training” in any sense. But this argument cannot be used —at least in the case that concerns us— as an excuse to make proposals that are unrealistic, to propose actions at an excessively large scale or solutions that omit any consideration of the environmental impact, or solutions that use unnecessary or disproportionate resources (e.g. in designs or in the use of materials)

Anyway, does the exercise of developing the structure for a major project, with an unconventional structural scheme, mean that students will be skilled enough to develop more conventional projects? We would seem to be faced with an example of the maxim \qui potest plus, potest minus” (“He who can do the most, can do less”): if someone has achieved an acceptable result for something that is highly complex, it seems evident that they have proven that they are able to do something that is “more normal”. Nevertheless, each different scale is associated with specific skills. Although it is true that in the degree course subjects learning these more “conventional” skill sets is assumed to have taken place, the master’s degree is distinguished by the fact that it functions at a “professional” level, so that documentation suitable for this level has to be produced. This differs very widely, depending on the size.

## Conclusions

- There are major differences between the structures planned in the master’s degree and those used in actual works.
- These differences arise in terms of size as well as constructive techniques, as student projects involve far larger constructions than the average-size buildings which are actually constructed. The majority of the former use constructive techniques that are very different from those used in conventional professional works.
- There is a need to reflect on whether the strategy of preparing master’s degree projects that are very different from more conventional buildings is suitable.
- Data show that there is no real concern about resource wastage.
- Related to the former point, teaching in architecture focuses on new projects, ignoring rehabilitation and reuse.

The teaching strategy which underlies the curriculum of the authorising master’s degree —and most particularly because it is authorising— consists of preparing a conceptual model that tries to represent a productive model [13]: in this case the preparation of a project that functions as a document for the construction of a building. The model will be useful if it fits the actual situation. If it does not, then it will have to be adapted to be appropriate for the real process by newly qualified architects when they start working.

The data analysed have made it possible to obtain clear conclusions. However, further analysis may be of interest, to discover whether any other tendencies exist.
